# Chronic Hepatitis B Infection Is Associated with Increased Molecular Degree of Inflammatory Perturbation in Peripheral Blood

**DOI:** 10.3390/v12080864

**Published:** 2020-08-07

**Authors:** Caian L. Vinhaes, Luís A. B. Cruz, Rodrigo C. Menezes, Thomas A. Carmo, María B. Arriaga, Artur T. L. Queiroz, Manoel Barral-Netto, Bruno B. Andrade

**Affiliations:** 1 Instituto Gonçalo Moniz, Fundação Oswaldo Cruz (FIOCRUZ), Salvador 40296-710, Brazil; caianleal@gmail.com (C.L.V.); rodrigocdemenezes@gmail.com (R.C.M.); thomasacarmo@gmail.com (T.A.C.); mbag711@gmail.com (M.B.A.); arturlopo@gmail.com (A.T.L.Q.); mbarralnetto@icloud.com (M.B.-N.); 2 Multinational Organization Network Sponsoring Translational and Epidemiological Research (MONSTER) Initiative, Salvador 41810-710, Brazil; luismedbcruz@gmail.com; 3 Curso de Medicina, Centro Universitário Faculdade de Tecnologia e Ciências (UniFTC), Salvador 41741-590, Brazil; 4 Faculdade de Medicina, Universidade Federal da Bahia, Salvador 40110-100, Brazil; 5 Curso de Medicina, Universidade Salvador (UNIFACS), Laureate Universities, Salvador 41720-200, Brazil; 6 Instituto Nacional de Ciencia e Tecnologia de Investigação Em Imunologia, São Paulo 05403-900, Brazil; 7 Escola Bahiana de Medicina e Saúde Pública (EBMSP), Salvador 40290-000, Brazil

**Keywords:** hepatitis B virus, inflammation, molecular degree of perturbation, biomarkers, viral load

## Abstract

Hepatitis B virus (HBV) infection remains a major public health concern. The interaction between HBV and the host inflammatory response is an important contributing factor driving liver damage and diseases outcomes. Here, we performed a retrospective analysis employing an adapted molecular degree of perturbation (MDP) score system to assess the overall inflammatory imbalance related to persistent HBV infection. Plasma levels of several cytokines, chemokines, and other inflammatory markers were measured in Brazilian individuals diagnosed with either chronic HBV or previous HBV infection, as well as in uninfected controls between 2006 and 2007. Multidimensional analyses were used to depict and compare the overall expression profile of inflammatory markers between distinct clinical groups. Chronic HBV patients exhibited a marked inflammatory imbalance, characterized by heightened MDP scores and a distinct profile of correlation networks inputting plasma concentrations of the biomarkers, compared with either individuals with previous HBV or controls. Furthermore, in participants with chronic HBV infection, the viral loads in peripheral blood were directly proportional to overall molecular perturbation as well as to specific perturbations of interleukin (IL)-4 and interferon (IFN)-γ concentrations. These findings highlight additional nuances about systemic inflammation related to persistent HBV infection.

## 1. Introduction

Hepatitis B virus (HBV) infection is one of the main concerns in public health worldwide. Overall, evidence of contact with the virus can be found in approximately two billion individuals [1]. Although there is a vaccine to protect humans against the virus, a number of 250 million patients with chronic disease was estimated in 2015 [2], and approximately 815,000 deaths were the result of HBV disease or its complications in 2016 [3]. Complications and outcomes in hepatitis B are directly influenced by inflammation and its associated tissue damage, often characterized by liver fibrosis, which can potentially lead to end-stage chronic liver diseases, such as cirrhosis and hepatocellular carcinoma [4,5,6].

HBV-associated tissue damage in the liver is quite well described and the host’s inflammatory response against infection is believed to be the most prominent cause associated with it, as the virus replication is not directly cytotoxic to the hepatocytes per se [4,5,6]. At the cellular level, the chronic spectrum of the disease is hallmarked by cycles of inflammation induced apoptosis, owing to maintained immune activity affecting infected hepatocytes, and tissue regeneration [1,4,6]. Furthermore, it has been reported that multiple cycles of hepatocyte injury and regeneration in different tissue locations play a part in the development of hepatocellular carcinoma [5,7]. The immune response associated with these cellular events is mainly characterized by predominant T-cell proliferation and exhaustion [8,9,10,11,12].

At the molecular level, higher release of serum hepatitis B surface antigen (HBsAg) in the circulation, expression of co-inhibitory receptors, and higher levels of interleukin (IL)-10 are thought to be associated with the cellular events described above [8,9,10,11,12,13,14,15]. However, the pathogenesis is yet to be completely understood. Recently, the influence of other factors driving the chronicity of the disease and the progress to its complications has been hinted, such as the regulation of microRNA expression and HBV DNA integration into the host genome [1,5,16]. Therefore, although chronic hepatitis B is well characterized by the cellular activity against infected hepatocytes, the disease setting and progression also appear to be closely related to innumerable molecular activities and its perturbations.

In the present study, using the molecular degree of perturbation (MDP) approach previously published by our group in a number of disease settings [17,18,19,20,21,22], a vast number of cytokines, chemokines, and acute phase proteins were analyzed from samples of chronic carriers of hepatitis B and of those with a history of natural HBV exposure, in order to identify differentially expressed markers associated with the immune pathogenesis of persistent HBV viral replication that may contribute to the high global burden disease.

## 2. Materials and Methods 

### 2.1. Ethics Statement

Written informed consent was obtained from all participants or their legally responsible guardians, and all clinical investigations were conducted according to the principles expressed in the Declaration of Helsinki. The project was approved by the institutional review board of the Faculdade de Medicina, Faculdade São Lucas, Rondônia, Brazil, where the study was performed (protocol No. 231465-05).

### 2.2. Study Design

The present study is based on analyses performed retrospectively in a databank containing clinical, immunological, and epidemiological data from 601 individuals recruited between 2006 and 2007 from the state of Rondônia, in the Brazilian Amazon, which is a highly endemic area for HBV infection. Multiple investigations have been reported from the project of which this study is part [23,24,25,26,27,28,29,30,31,32]. Patient investigation included passive case detection from individuals who sought care at the municipal hospital in Buritis (Rondônia, Brazil) and at the Brazilian National Foundation of Health (FUNASA) diagnostic centers, as well as active case detection in the municipalities of Buritis and Demarcação (Rondônia, Brazil). HBV diagnosis was conducted employing the AXSYM^®^ automatic ELISA system (Abbott, Wiesbaden, Germany); the evaluation included screening of HBsAg, hepatitis B e-Antigen (HBeAg), total anti-hepatitis B surface antigen (anti-HBs), total anti-hepatitis B core antigen (anti-HBc), anti-HBc IgM, and anti-hepatitis B e-Antigen (anti-HBe) IgG, and no acute HBV infection was detected. All the measurements were performed right at the study enrollment and at the diagnosis of current HBV infection, meaning that the collections were performed before the initiation of HBV-specific therapy. Viremia was estimated by real-time PCR (COBAS^®^ TaqMan^®^ HBV assay) of all samples to confirm serological results. For the present study, the exclusion criteria were as follows: documented viral hepatitis (A, C, and D); chronic alcoholism; human immunodeficiency virus type 1 infection; yellow fever; leptospirosis; cancer and chronic degenerative and inflammatory diseases; sickle cell trait and the use of hepatotoxic or immunosuppressant drugs and positive screening for malaria (microscopy), dengue (serology), and tuberculosis (clinical and/or radiological). Of note, all the HBV cases were first diagnosed at the time of the study enrollment and none were undertaking antiviral therapy. Thus, data from 601 patients were evaluated, including 29 patients with chronic HBV infection (HBsAg+, anti-HBS–, and anti-HBc+); from those, 18 were HBeAg+ and 11 were anti-HBe+, 64 patients with previous HBV infections (HBsAg–, anti-HBs+, and anti-HBc+) with undetectable HBV-DNA levels, and 83 uninfected healthy controls. Demographic and clinical characteristics of the study participants are described in Supplementary Tables S1 and S2.

### 2.3. Laboratory Measurements

Plasma levels of cytokines interleukin (IL)1-β, IL-4, IL-6, IL-10, IL-12p70, interferon (IFN)-γ, and tumor necrosis factor (TNF)-α, as well as of C-C motif chemokine ligand (CCL)2, CCL5, C-X-C motif chemokine ligand (CXCL)9, and CXCL10, were measured using the Cytometric Bead Array—CBA^®^ (BD Biosciences Pharmingen, San Diego, CA, USA), according to the manufacturer’s protocol. The measurements of aspartate amino-transferase (AST), alanine amino-transferase (ALT), total bilirubin, direct bilirubin, creatinine, fibrinogen, and C-reactive protein (CRP) were performed at the Pharmacy School of the Federal University of Bahia and at the clinical laboratory of Faculdade São Lucas.

### 2.4. Adaptation of Molecular Degree of Perturbation to Examine Plasma Concentrations of Biomarkers

The molecular inflammatory perturbation is based on the MDP method, used and recently published by our group [18,19,20]. In the present study, we inputted the plasma concentrations of a defined set of pre-selected biomarkers based on previously published studies from our group [23,24,25]. Thus, herein, the average plasma concentration levels and the standard deviation of a baseline reference group (uninfected controls group) were calculated for each biomarker. The MDP score of an individual biomarker was defined by taking the differences in concentration levels from the average of the biomarker in the reference group divided by the reference standard deviation. Essentially, the MDP score represents the difference, by number of standard deviations, from the healthy control group. The equation used to calculate MDP in the present study is shown below:

(1) $$Molecular\;degree\;of\;perturbation=\frac{x_{i}-{\bar{x}}_{\left(reference\right)}}{\sigma_{\left(reference\right)}}$$

(2) $$\sigma =\frac{\sum_{i=1}^{n}{\left(x_{i}-\bar{x}\right)}^{2}}{n-1}$$

*n* = Number of data points
*x_i_* = Each of the value of data
$\bar{x}$ = Mean of the data points
*σ* = Standard deviation

In this study, we applied the MDP scoring system using data on 20 biomarkers measured from three distinct groups of patients, with current HBV infection, previous HBV infection, and healthy uninfected controls. The MDP was filtered by the absolute MDP scores below two modules and by the sum of all accumulated MDP deviations. To identify samples implicated in “perturbation”, all values above the cutoff of the average MDP score plus two standard deviations of the reference group were considered “perturbed”.

### 2.5. Statistical Analysis

Descriptive statistics were performed to characterize the study population. Continuous variables were tested for Gaussian distribution using the D’Agostino–Pearson test. No variables exhibited normal distribution. The median values with interquartile ranges were used as measures of central tendency and dispersion, respectively. The Kruskal–Wallis test with Dunn’s multiple-comparison was used to compare continuous variables, whereas Pearson’s chi-square test was used to compare variables displayed as percentages. Hierarchical cluster analyses (Ward’s method) of log10 transformed and z-score normalized data were employed to depict the overall expression profile of indicated biomarkers in the study subgroups. All comparisons were pre-specified and two-tailed. Differences with *p*-values below 0.05 after Holm–Bonferroni’s adjustment for multiple comparisons were considered statistically significant. Profiles of correlation between biochemical parameters were examined using network analysis of the Spearman correlation matrices. Correlations with *p*-value < 0.05 were included in the network visualization. Spearman rank values (rho) were used to describe the strength of correlations between viral load from participants with current chronic infection and the MDP score of inflammatory parameters of these participants. The statistical analyses were performed using GraphPad Prism 8.0 (GraphPad Software, La Jolla, CA, USA), JMP 14.0 (SAS, Cary, NC, USA) and R statistical software.

## 3. Results

### 3.1. Characteristics of Participants

There were 176 participants in this study, who were grouped according to their exposure to HBV: 83 uninfected controls, 64 participants with previous HBV infection, and 29 chronic HBV carriers (18 HBeAg+ and 11 anti-HBe+). These participant groups were similar regarding biological sex and age (Supplementary Table S1). There was a high frequency of males in the current HBV group and females in the uninfected control group (Supplementary Table S1). Among the previous infected participants, we found an equal sex distribution (Supplementary Table S1).

### 3.2. Differences in Systemic Inflammation between the Study Subgroups

**Figure 1:**
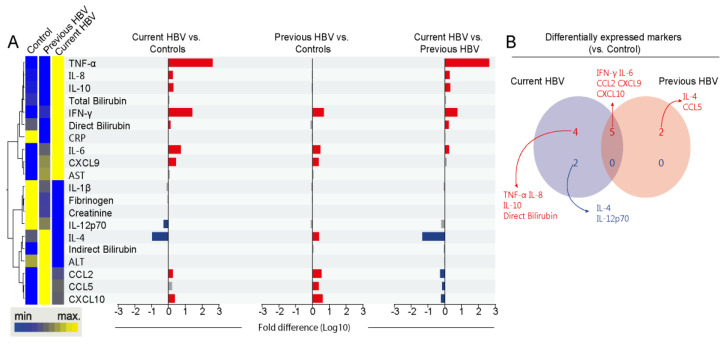
Systemic inflammatory profile of the study population. (**A**) Left panel: A hierarchical cluster analysis (Ward’s method) was employed to describe the overall expression pattern of plasma biomarkers in the study population. Dendrograms represent Euclidean distance. Right panel: Fold-differences in plasma levels of the indicated biomarkers between the study groups. Differences that reached statistical significance after adjustment for multiple comparisons (adjusted *p* < 0.05) are represented in colored bars. (**B**) Venn diagram demonstrates markers whose values were statistically different between each hepatitis B virus (HBV) group (current or previous infected) and healthy controls. Abbreviations (alphabetic order): ALT: alanine aminotransferase; AST: aspartate aminotransferase; CCL: C-C motif chemokine ligand; CXCL: C-X-C motif chemokine ligand; CRP: C-reactive protein; IFN: interferon; IL: interleukin; TNF: tumor necrosis factor.

To evaluate the systemic inflammatory profile in our study population, we conducted an unsupervised hierarchical cluster analysis of log-transformed and z-score normalized median concentration values of each biomarker per study group, which revealed three main clusters. The first cluster was characterized by increased levels of TNF-α, IL-6, IL-8, IL-10, total and direct bilirubin, IFN-γ, CRP, and CXCL9 in the current HBV group. The second cluster showed higher levels of IL-1β, fibrinogen, creatinine, and IL-12-p70 in the uninfected control group. Finally, the last cluster was composed by a higher expression of IL-4, indirect bilirubin, ALT, CCL2, CCL5, and CXCL10 in those with previous HBV infection (Figure 1A and Supplementary Table S2).

Then, we employed a Venn diagram composed of markers that exhibited statistically significant differences between the HBV groups versus uninfected controls (Figure 1B). Individuals with current HBV presented exclusively increased values of TNF-α, IL-6, IL-10, and direct bilirubin. IFN-γ, IL-6, CCL2, CXCL9, and CCL10 were shared with the group of individuals with previous HBV. Compared with the controls, the group with previous HBV showed exclusively higher values of IL-4 and CCL5. In addition, the group of patients with current HBV had lower IL-4 and IL-12p70 expression when compared with uninfected controls. These findings reinforce that HBV chronic carriers exhibit a distinct inflammatory profile activation.

### 3.3. HBV Infection is Associated with an Increased Degree of Inflammatory Perturbation

**Figure 2:**
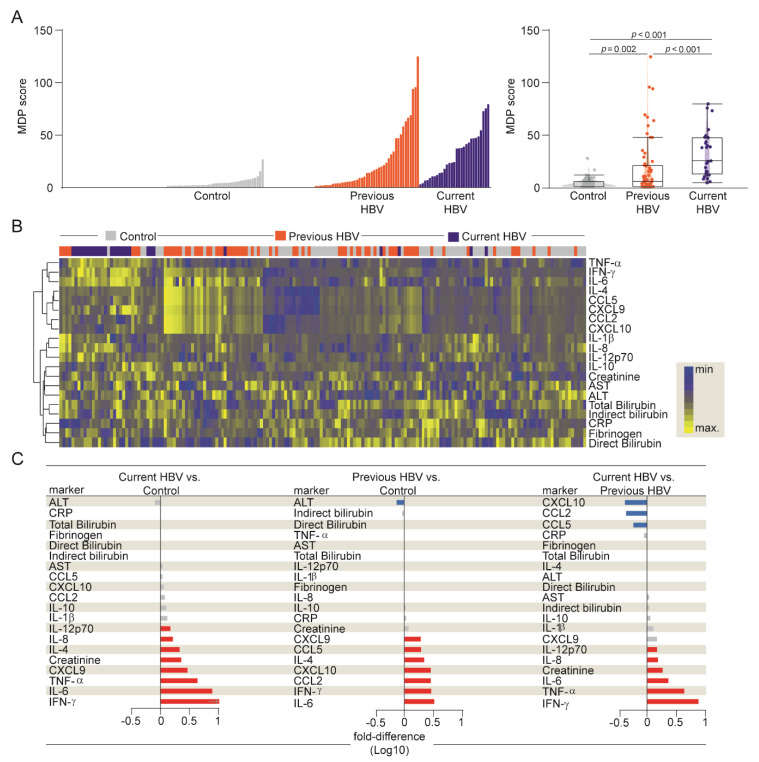
Higher degree of inflammatory perturbation associated with HBV infection. (**A**) Left panel: Histograms show the molecular degree of perturbation (MDP) score values relative to each study group, as indicated. Right panel: Box plots represent the MDP score distribution between study groups. Values were compared between patient groups using the Kruskal–Wallis test. (**B**) A hierarchical cluster analysis (Ward’s method) was employed to show the molecular degree of perturbation of each individual biochemical marker. (**C**) Average fold-difference values of the MDP scores for each marker were calculated between clinical groups. Red and blue bars represent statistically significant differences estimated by the Mann–Whitney *U* test. Abbreviations (alphabetic order): ALT: alanine aminotransferase; AST: aspartate aminotransferase; CCL: C-C motif chemokine ligand; CXCL: C-X-C motif chemokine ligand; CRP: C-reactive protein; IFN: interferon; IL: interleukin; TNF: tumor necrosis factor.

Then, we calculated the MDP score (as described in Methods) to directly test whether the differences in plasma concentrations found above would result in augmented overall inflammatory disturbance in HBV infection (Figure 2 and Supplementary Table S3). A similar approach has been recently employed by us to characterize systemic inflammation and activation, providing important insights into the pathophysiology of a variety of infectious diseases [17,18,19,20].

As expected, uninfected controls exhibited diminished MDP values, while participants with current HBV infection exhibited substantial increases in the median MDP score values when compared with controls and those with previous infection (Figure 2A and Supplementary Table S3). In addition, MDP scores of patients with previous disease were also significantly higher than those from uninfected controls (Figure 2A). Furthermore, we employed a hierarchical cluster analysis using MDP values calculated for each biomarker and found that the overall expression profile was distinct between the groups (Figure 2B and Supplementary Table S3).

Additional fold-difference analyses were used to quantify the magnitude of inflammatory disturbance between our three groups (Figure 2C). There was a general tendency towards a higher MDP score for each biomarker in the current HBV group. These participants expressed significantly higher perturbation scores of IL-12p70, IL-8, IL-4, creatinine, CXCL9, TNF-α, IL-6, and IFN-γ when compared with uninfected controls (Figure 2C). A similar tendency, with the same biomarkers, was observed when we extended our analysis to current versus previous HBV group, except for IL-4 and CXCL9 (Figure 2C). Importantly, the degree of perturbation in the levels of CXCL10, CCL2, and CCL5 was higher in previous HBV participants than in those with current disease. Moreover, previous HBV patients had higher MDP scores of CXCL9, IL-4, IL-6, and IFN-γ than the uninfected controls (Figure 2C). Our results argue that current HBV infection leads to an intense inflammatory imbalance, highlighting the markers implicated in HBV pathogenesis.

### 3.4. Network Analysis of Inflammatory Imbalance in Chronic HBV Infection

**Figure 3:**
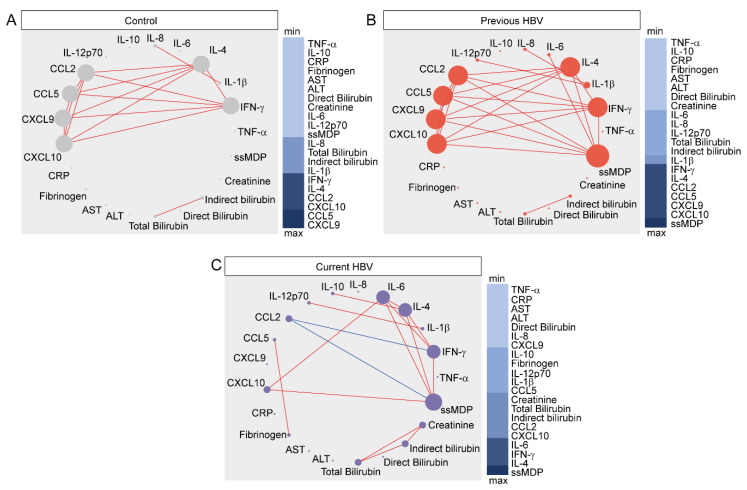
Network analysis of the MDP score matrices in the study groups. Spearman correlation matrices of the MDP score values in each study group ((**A**) controls; (**B**) previous HBV; (**C**) current HBV) were built and Circos plots were used to illustrate the correlation networks. Only significant correlations (*p* < 0.05) are shown. Each circle represents a different plasma parameter and the size of each circle is proportional to the number of significant correlations. Lines represent the Spearman rank (rho) values. Red color infers positive correlation, whereas blue color denotes negative correlation. Node analysis heatmap shows the number of statistically significant correlations involving each marker per clinical group. Abbreviations (alphabetic order): ALT: alanine aminotransferase; AST: aspartate aminotransferase; CCL: C-C motif chemokine ligand; CXCL: C-X-C motif chemokine ligand; CRP: C-reactive protein; IFN: interferon; IL: interleukin; TNF: tumor necrosis factor.

To further explore the nuances of the inflammatory perturbation of individual markers, as well as their direct effect on overall MDP values, we employed network analysis based on Spearman’s correlation matrices, as previously described [17,18,19,20] (Figure 3).

Thus, we used MDP scores for each biomarker, as well as the overall MDP score (ssMDP), and found that networks of distinct clinical groups exhibited differences in the complexity and quality of interactions between the levels of molecular perturbation. The highest density of significant correlations was found in group of previous HBV, whereas the lowest connectivity was detected in those with current infection (Figure 3).

Regardless of the clinical groups, most of the significant correlations were positive, meaning that increases in inflammatory perturbation of a given marker were mostly followed by increases in the disturbance levels of other inflammatory molecules. It is important to note that, in the uninfected control group, CXCL9 was the most connected marker, followed by CCL5 (Figure 3A). In the previous HBV group, the network analysis highlighted that the ssMDP was the most connected node and the markers that most contributed to its increases were CXCL10, CCL9, CCL5, CCL2, IL-4, and IFN-γ (Figure 3B). In patients with current HBV infection, our analyses showed that ssMDP was also the main marker, but with more strict influence of IL-4, IFN-γ, and IL-6 (Figure 3C). Importantly, CCL2, whose degree of perturbation exhibited only positive correlations in the group of previous HBV infection, displayed a negative correlation with the overall degree of perturbation and the IFNγ MDP score in the group of current HBV infection. These observations indicate that HBV chronic carrier patients exhibit a greater inflammatory perturbation, which also appears to be differentially regulated, with an uncoupling in the response, as there is a lower density of matrices and negative correlations.

### 3.5. Influence of HBV Viral Replication on the Systemic Inflammatory Imbalance

**Figure 4:**
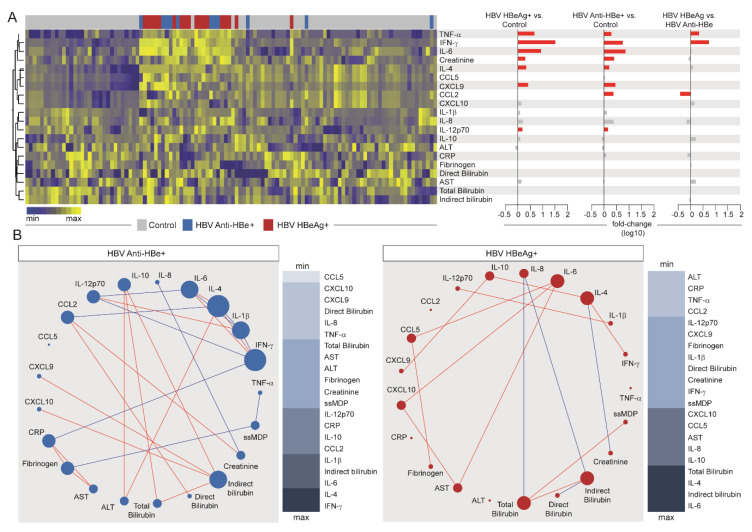
Effects of HBV viral replication on systemic inflammation. (**A**) Left panel: A hierarchical cluster analysis (Ward’s method) was employed to describe the overall expression pattern of plasma biomarkers in the study groups. Dendrograms represent Euclidean distance. Right panel: Fold-differences in plasma levels of the indicated biomarkers between the study groups. Differences that reached statistical significance after adjustment for multiple comparisons (adjusted *p* < 0.05) are represented in colored bars. (**B**) Spearman correlation matrices of the MDP score values in each study group were built and Circos plots were used to illustrate the correlation networks. Only significant correlations (*p*-value < 0.05) are shown. Each circle represents a different plasma parameter and the size of each circle is proportional to the number of significant correlations. Lines represent the Spearman rank (rho) values. Red color infers positive correlation, whereas blue color denotes negative correlation. Node analysis heatmap shows the number of statistically significant correlations involving each marker per clinical group. Abbreviations (alphabetic order): ALT: alanine aminotransferase; AST: aspartate aminotransferase; CCL: C-C motif chemokine ligand; CXCL: C-X-C motif chemokine ligand; CRP: C-reactive protein; IFN: interferon; IL: interleukin; TNF: tumor necrosis factor.

Our next step was to evaluate the influence of viral replication, assessed by HBeAg status, in the overall inflammatory profile among HBV chronic carrier patients (Figure 4 and Supplementary Table S4). Unsupervised hierarchical cluster analysis of log10-trasnformed values and z-score normalized MDP levels of each parameter revelaed no clear distinction of the inflammatory profile between those with active viral replication (HBeAg+) and those who were anti-HBe+, whereas both groups could be distinguished from the control participants (Figure 4A and Supplementary Table S4).

Fold change analyses (Figure 4A, right panel) revealed a similar pattern of inflammatory perturbations in HBeAg+ and Anti-HBe+ patients, when compared with the controls. Higher molecular perturbation of TNF-α, IFN- γ, IL-6, creatinine, IL-4, CXCL9, and IL-12p70 was found in those with chronic HBV infection, regardless of HBe status, whereas augmented perturbation in CCL2 was found in those with positive anti-HBe detection. When we extended our analysis to compare HBeAg+ versus anti-HBe+ participants, we found that only three markers were statistically different, TNF-α and IFN-γ (with higher levels of perturbation in those with active viral replication), and CCL2, which showed increased perturbation values in those with detectable anti-HBe (Figure 4A). These findings suggest that HBV chronic carriers present an inflammatory imbalance, regardless of the viral replication, and highlight that perturbation in levels of TNF-α and IFN-γ hallmark active HBV replication in such patients.

Spearman correlation matrices (Figure 4B) demonstrated that, in comparison with HBeAg+ patients, anti-HBe+ subjects had a greater number of significant correlations between the analyzed biomarkers. These correlations were predominantly positive as well. The top node in the correlation matrix from the group of anti-HBe+ patients was IFN-γ, which presented negative interactions with CRP, IL-12p70, and IL-1β, and positive correlations with IL-4 and IL-6. The following top node was IL-4, which presented negative correlations with CCL2 and IL-1β, and positive interactions with IL-6, IFN-β, and ALT. Of note, ssMDP score values were correlated only with TNF-α and fibrinogen in these patients. When the correlation profile of HBeAg+ patients was analyzed, the top connected node was IL-6, which presented positive correlations with CCL5, CXCL10, and AST. The following top node was indirect bilirubin, which presented negative correlations with IL-8 and direct bilirubin, and positive connection only with total bilirubin. In these patients, IL-4 presented positive significant correlations with IFN-γ and IL-10 and was negatively correlated to creatinine. The next top connected node was total bilirubin, which presented positive correlation with ssMDP and indirect bilirubin, as previously mentioned, and negative interaction with IL-8. These findings suggest that HBV replication, although not representing a huge impact on the systemic inflammatory imbalance, could lead to an uncoupling in the overall inflammatory response, translated by the detection of fewer connections in the correlation networks. These observations could also infer that the disease activity after the control of viral replication is mainly immune-driven.

**Figure 5:**
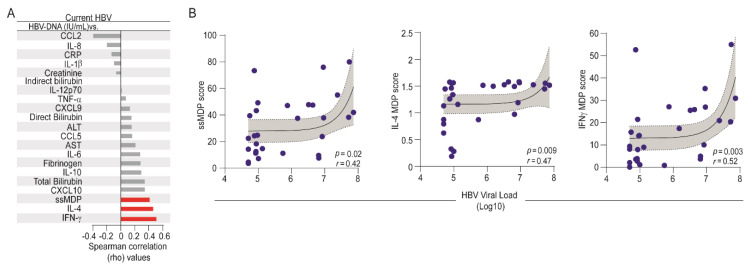
Associations between HBV-DNA viral loads and molecular degree of inflammatory perturbation. Spearman correlation analysis was employed to assess the correlations between the MDP score of each marker and HBV viral loads in patients with current HBV infection. (**A**) Bars show the rho values. Red bars infer the significant interactions (*p*-value < 0.05). (**B**) Lines represent linear adjustment of the curve with 95% confidence intervals for each parameter that was significantly correlated with HBV viral load. Abbreviations (alphabetic order): ALT: alanine aminotransferase; AST: aspartate aminotransferase; CCL: C-C motif chemokine ligand; CXCL: C-X-C motif chemokine ligand; CRP: C-reactive protein; IFN: interferon; IL: interleukin; TNF: tumor necrosis factor.

Finally, we performed an additional Spearman correlation analysis to directly evaluate the associations between the HBV-DNA viral loads and the degree of inflammatory perturbation in HBV chronic carriers (Figure 5). We found that the HBV viral load was directly associated with ssMDP score values (*r* = 0.42; *p* = 0.02) as well as with the degree of perturbation of IL-4 (*r* = 0.47; *p* = 0.009) and IFN-γ (*r* = 0.52; *p* = 0.003) levels in plasma (Figure 5A,B). These findings identified the specific markers that were influenced by HBV viremia.

## 4. Discussion

In the present study, we analyzed multiple key biomarkers of the HBV-associated immune response. To properly examine their role in the context of inflammation in HBV infections, we applied an adaptation of the molecular degree of perturbance concept, which, in summary, reflects the distance between the levels of a biomarker, from its expression in healthy state. This approach allows a more intrinsic evaluation of the inflammatory pathways involved in diseases. We were able to demonstrate that patients with chronic hepatitis B indeed present an increased molecular degree of inflammatory perturbation imposed by the infection, with results that provide deeper insights into the host’s systemic response against the hepatitis B virus in the context of active viral replication.

The evaluation of chronic HBV patients revealed a particular inflammatory profile, when compared with the uninfected controls and those previously exposed to HBV. This profile was notably marked by pronounced elevations of TNF-α, IFN-γ, and IL-6, as well as significant, but subtle, increased plasma concentrations of IL-10 and IL-8 biomarkers. These results indicate a pro-inflammatory imbalance in these patients, which may be induced by viral persistence. Of note, the contrast between IFN-γ and IL-10 levels has also been correlated with worse clinical presentations in other diseases such as tuberculosis and malaria [24,26,33]. Indeed, immune responses to chronic HBV infections have been extensively associated with a sustained pro-inflammatory state and with antiviral responses related to IFN-γ activity [1,4,6]. In contrast to this condition, acute resolving hepatitis B has been associated with coordinated immune responses [5,34]. Interestingly, in our investigation, patients who were previously infected with HBV maintained high circulating levels of CCL2, CCL5, and CXCL10, which are chemokines that display a fundamental role in the maintenance of both innate and adaptive responses [35]. CXCL10 is an IFN-γ induced protein and alterations in its expression have been associated with inflammatory milieu and infectious diseases [36], whereas CCL5 has been reportedly associated with a crucial role in hepatocellular carcinoma development in murine and human models [37]. CCL2, which is an important chemokine involved in recruitment of natural killer (NK) cells [38] and monocytes [38,39], had its productions associated especially with hepatocytes under acute HBV-driven stress [40]. In addition, previous studies have also inferred its influence over CD4^+^ T lymphocyte function towards a more IL-4 biased response [41]. Importantly, CCL2, CCL5, and CXCL10 have been described in the process of conversion of senescent premalignant cells mediated by oncogenes activation that could culminate with hepatocellular carcinoma [42,43,44]. Summarizing, these results potentially indicate that, despite the tendency of a more favorable outcome after immune-clearance of HBV-DNA levels [45], these patients remains with increased levels of perturbation from several inflammatory markers. Our findings raise relevant points that could infer a possibility of hepatocellular carcinoma development even after the viral clearance, highlighting and reinforcing the necessity of continued care from previous HBV carriers. Future prospective studies are necessary to properly answer these questions.

Immune activation after an infectious trigger is a complex process that occurs with synchronized changes and interactions between inflammatory mediators. Using Spearman’s correlation matrices, we have extensively studied the quality and magnitude of inflammation in several disease models [17,20,25,46]. The uninfected control group presented some interesting correlation results. Although no other active infectious disease or inflammatory condition could be diagnosed at study enrollment, these results might be influenced from past diseases typically found on the region at the time, which have been reported in this population, such as previous malaria episodes, for example [26]. Notably, in the uninfected control group, the ssMDP showed no significant connections, suggesting that no marker was influencing an overall disturbance of inflammation, reflecting the homeostasis. In contrast, in HBV patients, either currently or previously infected, the ssMDP was the most connected node, inferring that changes in levels of cytokines and chemokines are driving dissimilarities in systemic inflammation. Of note, a higher density of connections was observed in the matrices of participants with previous HBV infections. Moreover, in consonance with previous analysis, ssMDP correlations with CCL2, CCL5, and CXCL9 MDP scores were those hallmarked by significant differences in this group. Furthermore, all these chemokines were also mainly linked to the node of IL-4. Hence, these results may suggest some degree of reordination in the immunological response, despite the maintenance of perturbation, and also that this residual imbalance may be a consequence of the interactions between both the activation of the innate and adaptative immune response against the virus. Curiously, correlation matrices of chronic HBV patients exhibited an increased perturbation pattern with lower density connections, also remarkable by significant negative interactions displayed in the network analysis, indicating an uncoupling in inflammatory responses. These negative correlations were related to the CCL2 node, in contrast to the biosignature presented by patients previously infected by HBV. As shown in Supplementary Table S3 and Figure 2, patients with current HBV infections exhibited significantly elevated IFN-γ and reduced CCL2 MDP scores. Therefore, it can be interpreted that ssMDP is associated with distinct inflammatory mechanisms in patients with past and resolved HBV infections, and in those with active chronic HBV, which present a more pronounced pro-inflammatory expression. Certainly, these results reveal a novel improvement over the current knowledge about inflammatory response in persistent HBV infections, providing relevant insights about the differential molecular profile regulation and pathogenesis in this group. Finally, our findings could also represent how the inflammatory imbalance, with aggressive, but ineffective responses, is associated with viral persistence and disease progression, possibly until the life-threatening liver damage setting.

The next step was to analyze the influences of the viral replication in the inflammatory activation in HBV. Considering the persistence of inflammatory imbalance responses in HBeAg+ and anti-HBe+ subjects, our results highlight the presence of a sustained molecular perturbation profile in both groups that is mainly indifferent to the replication status. Despite no clear dissimilarities being observed, both hierarchical cluster and fold change analysis depict the maintenance of the heightened perturbation state among those with active and controlled viral replication. Thus, reinforcing that, regardless of the replication activity, several markers cited previously, such as TNF-α, IFN-γ, and IL-6, remained significantly inflecting overall inflammatory responses of the group of current HBV patients. Noteworthy, the distinguishable patterns presented by the correlation matrices also suggest that the fewer number of significant connections observed in the HBeAg+ group may comprise a differential inflammatory interaction that can have an influence over the disease immunopathogenesis during replication activity. Of note, the lower density of interactions displayed at the network analysis also points to a potential disarrangement of the inflammatory response in this state. After identifying these dissonances in the immune response associated with viral persistence and the establishment of chronic disease, our further analysis focused on the impact of viral load in the degree of inflammatory perturbance in chronic HBV. Our results demonstrated that IFN-γ and IL-4 MDP scores were associated with higher levels of HBV-DNA. These findings, in agreement with previous studies, reflect the ineffectiveness of immune response in the combat to HBV in those with chronic infection, with a tendency of higher IFN-γ and IL-4 disturbance associated with a more pronounced viral load [47]. Additionally, the overall expression of perturbation also showed a positive correlation with the HBV viral load. As the serum HBV-DNA levels are associated with an increased risk of fibrosis and hepatocellular carcinoma [48], our results may reinforce the idea of an association between inflammatory imbalance and unfavorable outcomes in chronic HBV infections.

Our study has some limitations. We did not have data available from follow-up of the HBV-infected patients, and for this reason, additional information on clinical evolution, sequential ALT measurements, and implementation of antiviral treatment after blood collection were not obtained. The individuals who were diagnosed with HBV infection were referred to a specialized service, which meant the follow up could not be embraced by our project at that time, and made it inviable to evaluate the inflammatory interplay over time, as well as clinical outcomes such as cirrhosis development in the participants with persistent infection. Regardless of such limitations, our study expands current information regarding HBV infections, and provides relevant insights into the role of general inflammatory activation in HBV infection. Our focus using the MDP was to try to gather additional insights about mechanisms of pathophysiology in HBV infection. We judge our findings as relevant because they contribute to our understanding about the regulation of systemic inflammatory pathways, which can be read through our network analysis. If the results presented here are validated by other cohorts, this concept of deconvoluting disease pathogenesis to measurable metrics such as MDP or network density could be merged with clinical outcomes to result in a more global understanding of the mechanisms underlying susceptibility to infection of even disease tolerance in the context of HBV infection. Taken together, such concepts could be translated into personalized medicine in which important cytokines identified to be linked to disease susceptibility could be pharmacologically modulated.

## 5. Conclusions

Our findings reveal that patients with persistent HBV infection exhibit a distinct biosignature of inflammatory markers, with higher levels of immune disturbance that characterize the HBV chronic carriers. The analyses indicate that HBV viral load directly influences the overall degree of inflammatory perturbation, which was closely related with perturbation in IFN-γ and IL-4 levels. Additionally, the results also indicate that there is a potential residual hyperinflammatory milieu in participants with previous HBV infection, reinforcing the necessity of continuate care.

## Supplementary Materials

The following are available online at https://www.mdpi.com/1999-4915/12/8/864/s1, Table S1: Characteristics of study participants; Table S2: Biochemical evaluation of clinical groups; Table S3: Molecular perturbation score for each marker between clinical groups; Table S4: Biochemical evaluation of HBeAg+, anti-HBe+ and control participants.
