# Supplementary material for: Chronic Hepatitis B Infection Is Associated with Increased Molecular Degree of Inflammatory Perturbation in Peripheral Blood
Source: Viruses. 2020 Aug 7;12(8):864. doi: 10.3390/v12080864 (PMC7472740; doi:10.3390/v12080864)
Supplement: Supplementary file 1 [file viruses-12-00864-s001.pdf]

**Table S1. Characteristics of study participants.**

| Characteristic | Healthy controls | Previous HBV | Current HBV | P-value |
|---|---|---|---|---|
| N | 83 | 64 | 29 | |
| Age – y | 40 (26-53) | 35 (25-48.7) | 38 (23-50) | 0.34 |
| Male – no. (%) | 37 (44) | 32 (50) | 16 (55) | 0.58 |

Data represent medians and interquartile ranges (age) and frequencies (male gender). The Kruskal-Wallis test was used to compare distributions of age while the Chi-square test was used to compare frequencies. P-values in bold font are statistically significant.

## Table S2. Biochemical evaluation of clinical groups.

| Parameter | unit | Healthy Controls | Previous HBV | Current HBV | P-value |
|---|---|---|---|---|---|
| N | | 83 | 64 | 29 | |
| IL-1β | pg/mL | 5.95 (3.75-17.75) | 5.26 (3.60-10.63) | 4.75 (1.18-15.20) | 0.5708 |
| IL-4 | pg/mL | 16.81 (11.29-7.51) | 40.01 (28.99-52.17) | 1.83 (0.63-9.85) | **<0.001** |
| IL-6 | pg/mL | 6.50 (3.70-9.40) | 19.50 (7.0-29.40) | 35.50 (22.0-46.15) | **<0.001** |
| IL-8 | pg/mL | 6.06 (4.60-9.97) | 5.74 (4.79-9.75) | 11.12 (7.03-17.66) | **0.009** |
| IL-10 | pg/mL | 12.0 (6.8-19.0) | 11.0 (7.0-17.88) | 23.0 (15.80-33.95) | **<0.001** |
| IL-12p70 | pg/mL | 7.64 (4-65-16.11) | 6.3 (4.2-10.85) | 3.9 (1.81-12.31) | **0.03** |
| TNF-α | pg/mL | 0 (0-5.9) | 0 (0-8.3) | 43.10 (28.45-56.45) | **<0.001** |
| IFN-γ | pg/mL | 12.30 (0-28.0) | 57.55 (37.98-83.75) | 321.0 (100.4-570.8) | **<0.001** |
| CCL2 | ng/mL | 52.63 (18.59-96.06) | 182.0 (124.1-255.9) | 96.81 (46.41-167.1) | **<0.001** |
| CCL5 | ng/mL | 20,169 (14,215-3,483) | 46,584 (33,753-0,736) | 32,650 (17,889-4,330) | **<0.001** |
| CXCL9 | ng/mL | 206.0 (145.5-341.8) | 470.50 (340.9-612.8) | 583.70 (220.1-1,467) | **<0.001** |
| CXCL10 | ng/mL | 52.44 (9.90-106.7) | 214.10 (141.70-6.50) | 126.0 (78.23-162.10) | **<0.001** |
| Fibrinogen | mg/dL | 222.50 (198.30-293.6) | 207.90 (187.9-297.9) | 203.50 (176.2-240.5) | 0.347 |
| CRP | ng/mL | 4.80 (4.0-7.50) | 4.50 (3.53-5.88) | 4.80 (4.0-6.75) | 0.202 |
| Creatinine | mg/dL | 1.24 (1.09-1.29) | 1.21 (1.04-1.31) | 1.20 (0.83-1.46) | 0.912 |
| AST | U/L | 37.90 (33.40-47.50) | 43.20 (33.75-54.30) | 45.0 (34.15-55.50) | 0.169 |
| ALT | U/L | 38.80 (32.20-47.20) | 39.20 (33.98-44.88) | 37.40 (33.45-43.45) | 0.902 |
| Total Bilirubin | mg/dL | 0.70 (0.50-1.0) | 0.68 (0.48-1.0) | 0.80 (0.54-1.15) | 0.355 |
| Direct Bilirubin | mg/dL | 0.30 (0.19-0.40) | 0.23 (0.12-0.34) | 0.40 (0.30-0.50) | **0.008** |
| Indirect Bilirubin | mg/dL | 0.40 (0.28-0.65) | 0.46 (0.30-0.62) | 0.40 (0.27-0.62) | 0.614 |
| HBVDNA | IU/mL | - | - | 23571 (14232-1332146) | |

Data represent medians and interquartile ranges. The Kruskal-Wallis test was used to compare the distributions of the plasma mediators between the study groups. P-values in bold font are statistically significant.

**Table S3. Molecular perturbation score for each marker between clinical groups.**

| Parameter | unit | Healthy Controls | Previous HBV | Current HBV | P-value |
|---|---|---|---|---|---|
| N | | 83 | 64 | 29 | |
| IL-1β | MDP Score | 0.52 (0.41-0.83) | 0.53 (0.44-0.71) | 0.71 (0.46-0.82) | 0.70 |
| IL-4 | MDP Score | 0.65 (0.35-0.95) | 1.48 (0.82-2.41) | 1.44 (0.92-1.53) | **<0.001** |
| IL-6 | MDP Score | 0.43 (0.20-1.0) | 1.5 (0.34-2.78) | 3.6 (1.82-5.0) | **<0.001** |
| IL-8 | MDP Score | 0.50 (0.33-0.96) | 0.53 (0.38-0.70) | 0.85 (0.53-1.83) | **0.01** |
| IL-10 | MDP Score | 0.60 (0.31-1.0) | 0.66 (0.28-0.91) | 0.78 (0.27-1.79) | 0.73 |
| IL-12p70 | MDP Score | 0.65 (0.38-0.90) | 0.65 (0.32-0.82) | 1.0 (0.67-1.11) | **0.009** |
| TNF-α | MDP Score | 0.57 (0.36-0.57) | 0.57 (0.43-0.57) | 2.6 (1.5-3.6) | **<0.001** |
| IFN-γ | MDP Score | 0.59 (0.34-0.90) | 1.77 (0.91-3.0) | 14.0 (3.77-25.7) | **<0.001** |
| CCL2 | MDP Score | 0.58 (0.35-0.90) | 1.74 (0.96-2.91) | 0.73 (0.36-1.5) | **<0.001** |
| CCL5 | MDP Score | 0.71 (0.37-1.0) | 1.43 (0.80-2.38) | 0.80 (0.47-1.4) | **<0.001** |
| CXCL9 | MDP Score | 0.72 (0.36-1.0) | 1.44 (0.80-2.39) | 2.2 (0.51-8.0) | **<0.001** |
| CXCL10 | MDP Score | 0.58 (0.35-0.90) | 1.74 (0.96-2.91) | 0.70 (0.42-1.0) | **<0.001** |
| Fibrinogen | MDP Score | 0.58 (0.46-1.0) | 0.60 (0.47-1.1) | 0.60 (0.24-1.0) | 0.75 |
| CRP | MDP Score | 0.55 (0.32-0.86) | 0.62 (0.42-0.82) | 0.53 (0.30-0.86) | 0.62 |
| Creatinine | MDP Score | 0.56 (0.33-0.88) | 0.70 (0.33-0.97) | 1.34 (0.74-2.78) | **<0.001** |
| AST | MDP Score | 0.66 (0.31-1.0) | 0.66 (0.30-1.0) | 0.74 (0.32-1.2) | 0.63 |
| ALT | MDP Score | 0.61 (0.32-1.0) | 0.44 (0.18-0.71) | 0.47 (0.21-0.76) | 0.05 |
| Total Bilirubin | MDP Score | 0.75 (0.43-1.3) | 0.75 (0.44-1.0) | 0.73 (0.50-1.3) | 0.94 |
| Direct Bilirubin | MDP Score | 0.66 (0.20-1.25) | 0.61 (0.32-1.0) | 0.66 (0.29-1.2) | 0.87 |
| Indirect Bilirubin | MDP Score | 0.74 (0.37-1.0) | 0.65 (0.30-0.98) | 0.74 (0.24-1.4) | 0.23 |

Data represent medians and interquartile ranges. The Kruskal-Wallis test was used to compare the distributions of the plasma mediators between the study groups. P-values in bold font are statistically significant.

**Table S4. Biochemical evaluation of HBeAg+, Anti-HBe+ and control participants.**

| Parameter | unit | Healthy Controls | HBeAg+ | Anti-HBe+ | P-value |
|---|---|---|---|---|---|
| N | | 83 | 18 | 11 | |
| TNF-α | MDP Score | 0.58 (0.36 - 0.58) | 2.84 (2.24 - 3.77) | 1.21 (0.58 - 2.99) | < **0.001** |
| IFN-γ | MDP Score | 0.59 (0.34 - 0.9) | 20.63 (9.36 - 27.11) | 3.56 (1.1 - 14.24) | < **0.001** |
| IL-1β | MDP Score | 0.52 (0.41 - 0.83) | 0.67 (0.45 - 0.9) | 0.72 (0.48 - 0.83) | 0.853 |
| IL-4 | MDP Score | 0.65 (0.35 - 0.96) | 1.51 (1.19 - 1.55) | 1.13 (0.62 - 1.46) | < **0.001** |
| IL-6 | MDP Score | 0.44 (0.2 - 1.05) | 4.03 (2.66 - 5.25) | 3.43 (0.32 - 4.1) | < **0.001** |
| IL-8 | MDP Score | 0.5 (0.33 - 0.97) | 0.85 (0.61 - 1.18) | 1.3 (0.32 - 2.5) | 0.052 |
| IL-10 | MDP Score | 0.6 (0.32 - 1) | 0.81 (0.28 - 1.93) | 0.46 (0.27 - 1.66) | 0.551 |
| IL-12p70 | MDP Score | 0.65 (0.38 - 0.91) | 1.05 (0.56 - 1.81) | 1 (0.81 - 1.09) | **0.003** |
| CCL2 | MDP Score | 0.59 (0.35 - 0.9) | 0.54 (0.29 - 0.83) | 1.48 (0.82 - 3.02) | **0.005** |
| CCL5 | MDP Score | 0.72 (0.37 - 1.03) | 0.78 (0.51 - 1.34) | 0.84 (0.36 - 1.48) | 0.436 |
| CXCL9 | MDP Score | 0.72 (0.36 - 1.02) | 1.98 (0.52 - 6.8) | 2.2 (0.52 - 9.39) | **0.002** |
| CXCL10 | MDP Score | 0.59 (0.35 - 0.9) | 0.87 (0.46 - 1.28) | 0.57 (0.1 - 0.83) | 0.139 |
| CRP | MDP Score | 0.56 (0.33 - 0.87) | 0.5 (0.28 - 0.74) | 0.71 (0.45 - 1.03) | 0.260 |
| Fibrinogen | MDP Score | 0.59 (0.46 - 1.09) | 0.65 (0.16 - 1.06) | 0.55 (0.42 - 1.1) | 0.901 |
| AST | MDP Score | 0.66 (0.31 - 1.05) | 0.99 (0.65 - 1.26) | 0.56 (0.27 - 1.05) | 0.210 |
| ALT | MDP Score | 0.62 (0.33 - 1.03) | 0.46 (0.25 - 0.79) | 0.48 (0.19 - 0.6) | 0.305 |
| Total Bilirubin | MDP Score | 0.76 (0.43 - 1.33) | 0.76 (0.46 - 1.36) | 0.73 (0.55 - 1.06) | 0.837 |
| Direct Bilirubin | MDP Score | 0.67 (0.21 - 1.25) | 0.67 (0.5 - 1.25) | 0.67 (0.08 - 1.25) | 0.891 |
| Indirect bilirubin | MDP Score | 0.74 (0.37 - 1.07) | 0.68 (0.25 - 1.4) | 0.82 (0.37 - 1.48) | 0.761 |
| Creatinine | MDP Score | 0.57 (0.34 - 0.88) | 1.2 (0.75 - 3.06) | 1.54 (0.75 - 2.65) | < **0.001** |

Data represent medians and interquartile ranges. The Kruskal-Wallis test was used to compare the distributions of the plasma mediators between the study groups. P-values in bold font are statistically significant
